# Optical Gas Sensing with Liquid Crystal Droplets and Convolutional Neural Networks

**DOI:** 10.3390/s21082854

**Published:** 2021-04-18

**Authors:** José Frazão, Susana I. C. J. Palma, Henrique M. A. Costa, Cláudia Alves, Ana C. A. Roque, Margarida Silveira

**Affiliations:** 1 Institute for Systems and Robotics (ISR), Instituto Superior Técnico (IST), University of Lisbon, 1049-001 Lisbon, Portugal; jose.l.frazao@tecnico.ulisboa.pt; 2 UCIBIO, Chemistry Department, NOVA School of Science and Technology, Universidade NOVA de Lisboa, 2829-516 Caparica, Portugal; s.palma@fct.unl.pt (S.I.C.J.P.); hma.costa@campus.fct.unl.pt (H.M.A.C.); cma.alves@campus.fct.unl.pt (C.A.); cecilia.roque@fct.unl.pt (A.C.A.R.)

**Keywords:** liquid crystal, gas sensing, volatile organic compound, CNN, LSTM, YOLO

## Abstract

Liquid crystal (LC)-based materials are promising platforms to develop rapid, miniaturised and low-cost gas sensor devices. In hybrid gel films containing LC droplets, characteristic optical texture variations are observed due to orientational transitions of LC molecules in the presence of distinct volatile organic compounds (VOC). Here, we investigate the use of deep convolutional neural networks (CNN) as pattern recognition systems to analyse optical textures dynamics in LC droplets exposed to a set of different VOCs. LC droplets responses to VOCs were video recorded under polarised optical microscopy (POM). CNNs were then used to extract features from the responses and, in separate tasks, to recognise and quantify the vapours exposed to the films. The impact of droplet diameter on the results was also analysed. With our classification models, we show that a single individual droplet can recognise 11 VOCs with small structural and functional differences (F1-score above 93%). The optical texture variation pattern of a droplet also reflects VOC concentration changes, as suggested by applying a regression model to acetone at 0.9–4.0% (*v/v*) (mean absolute errors below 0.25% (*v/v*)). The CNN-based methodology is thus a promising approach for VOC sensing using responses from individual LC-droplets.

## 1. Introduction

**Figure 1:**
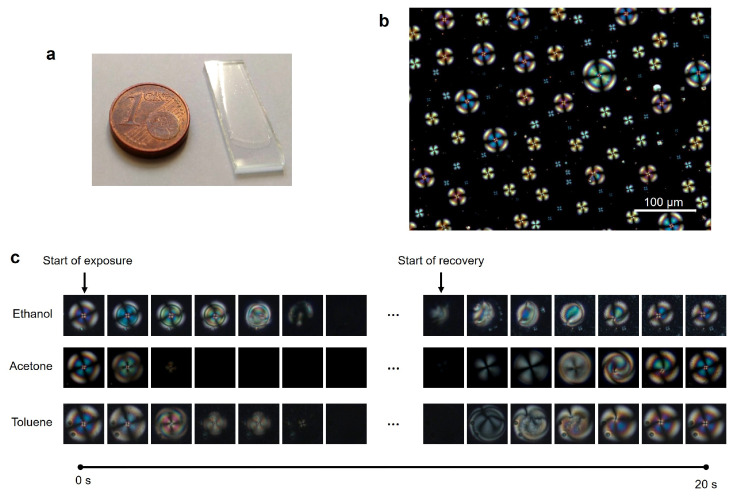
Macroscopic and microscopic appearance of the hybrid gel. (**a**) Hybrid gel spread as a thin film over a glass slide. (**b**) Polarising optical microscopy image, taken with crossed polarisers, of a hybrid gel region before VOC exposure. (**c**) Evolution of the optical texture of LC droplets during an exposure/recovery cycle with ethanol, acetone or acetone vapours.

The study of liquid crystals as platforms for gas sensing attracts increasing interest from the scientific community due to their fast and reversible optical responses, low energy-demand, operation at room temperature and tunable selectivity [1]. These are advantages when compared to the conventional semiconductor metal oxide [2] and polymeric [3] gas sensors, that require high operating temperatures and lack selectivity. These LC properties are valuable to develop new portable and low-cost devices for real-time detection of odours and volatile organic compounds (VOC), which can find applications in fields like industrial manufacture [4] and food quality control [5], air quality monitoring [6] and medical diagnostics [7,8,9,10]. LC are anisotropic materials that possess self-assembly properties and orientational molecular order. When LC molecules are orderly aligned along an axis, they can rotate the plane of polarised light, allowing light transmission through the material and generating interference patterns and colours (optical textures) observable under polarised optical microscopy (POM). Perturbation of molecular order results in changes in the optical textures and consequently light transmission. This property is the basis for using LC materials for biological and chemical sensing [11,12,13], including analytes in the gas phase [1,14,15]. Typically, the LC molecules have a specific orientation at a given support or interface and the interaction with volatile organic compound (VOC) molecules disturbs that orientation. In general, the VOC interaction monitoring is done by polarising optical microscopy observations of the materials [16,17] without automated analysis, or by measuring the variations of light transmission of the materials throughout time [5,18,19,20], which corresponds to 1D signals. More recently, approaches based on artificial intelligence methods started to be employed to automatically identify VOCs based on features of the LC responses [20,21,22]. Namely, we showed that a support vector machines classifier (SVM) based on features manually extracted from the 1D optical signal generated by hybrid gels containing LC spherical droplets embedded in a gelatin matrix (Figure 1a) accurately classified VOCs from distinct chemical classes [20]. In hybrid gels, the presence of ionic liquid at the interface between gelatin and the droplets results in LC radial configuration (Figure 1b). In the presence of different VOCs the LC radial configuration is gradually disturbed and recovered when the VOC is removed (Figure 1c). VOCs with different chemical functionalities interact preferentially with different components of the gel. As a result of the distinct interactions, LC orientational changes happen in different timings and patterns, generating distinct dynamic optical texture variations, as previously reported [19].

The optical texture variations constitute interesting and information-rich 2D signals along time. Thus, an alternative to using the 1D optical signal for VOC identification would be the application of image recognition methods that could automatically learn the optical textures time patterns associated to each VOC. Convolutional neural networks (CNN) are suitable for this type of analysis, and can fully use the data richness of image sequences without the need for manual feature extraction. So far the use of CNNs to analyse liquid crystal-VOC interaction 2D data was only reported for sequences of images obtained from a planar LC sensor [21,22]. Cao et al. [21] demonstrated that a CNN algorithm applied to grayscale sequences of polarised optical microscopy images could improve the selectivity of a planar LC-based sensor for water vapour and the VOC dimethyl-methylphosphonate (DMMP). However, the time domain information was not fully explored since only 2D CNN were used. Cao et al. [21] used Alexnet [23], a well-known object recognition CNN, but only to extract 2D features from individual frames in the videos that were acquired. Then, these features were combined for all the frames in the sequence and used for classification with an SVM. Smith et al. [22] used the same dataset but explored the colors of the images and used a more compact 2D CNN, the VGG16 [24], which allowed to reduce the number of features with similar classification accuracy of DMMP.

In the past, we demonstrated that the 1D signal corresponding to the intensity of polarised light transmitted through hybrid gels contains VOC distinctive features [5,19,20]. Here, we developed a pattern recognition system based on CNN video analysis of the configurational changes of LC droplets in a hybrid gel upon exposure to VOC samples. Using information of individual droplets, the system successfully performed two distinct tasks. In the first task, it accurately classified 11 VOCs representative of distinct chemical classes (alkanes, alcohols, ketones, aromatics, chlorinated compounds). In the second task, the system predicted the concentration of acetone vapours presented to the gel. These results indicate that each VOC generates a typical and reproducible optical pattern at the single droplet level. Naturally, the pattern varies also with the number of molecules presented to the droplets. Our pattern recognition method does not require manual feature engineering and fully exploits space and time information in the videos. The fast response (less than 20 s) of single droplet responses combined with the success of CNN-based approaches and the gel nature of the sensing material evidence the potential of the system towards miniaturised and automated gas sensors, compatible with flexible substrates and mouldable in different shapes.

## 2. Materials and Methods

### 2.1. Reagents

Gelatin from bovine skin (gel strength 225 g; bloom, type B) was purchased from Sigma-Aldrich (Darmstadt, Germany), the liquid crystal 4-cyano-4′-pentylbiphenyl (5CB) was purchased from TCI Europe (Zwijndrecht, Belgium), and the ionic liquid [BMIM] [DCA] (≥98%) was purchased from IoLiTec (Heilbronn, Deutschland). The solvents dichloromethane and hexane were purchased from VWR (Radnor, United States), and ethanol (purity ≥99.8%) was purchased from Sigma-Aldrich. Acetonitrile (purity ≥99.9%), chloroform, diethyl ether (HPLC grade), diethyl acetate, heptane, methanol (HPLC grade), and toluene were supplied by Fisher Scientific (Hampton, VA, USA). Acetone (purity ≥99.5%) was purchased from Honeywell (Charlotte, NC, USA). Solvents were of analytical grade and used as received.

### 2.2. Video-Recording of the Sensor’s Optical Response to VOCs

**Figure 2:**
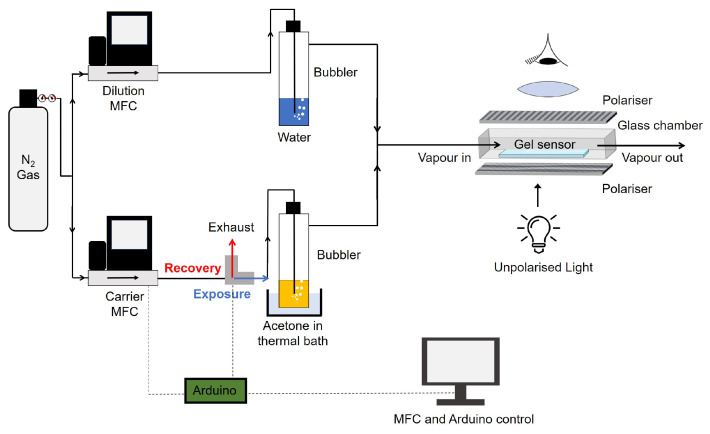
Gas delivery system used to generate controlled concentrations of acetone vapours and send them to the sensor chamber positioned in a polarised optical microscope. Two mass flow controllers (MFC) allow to mix acetone and humid nitrogen vapours in different proportions, and the electro-valve (in grey) switches between exposure and recovery stages. The MFCs and electro-valve are controlled by an Arduino microcontroller.

Gelatin hybrid gels spread as thin films (30 μm thickness) over untreated glass slides were used as VOC optical sensors, following procedures previously described [19,20]. The hybrid gel sensors were positioned inside a glass chamber placed in the stage of a polarising optical microscope (Zeiss Axio Observer. Z1/7, (Zeiss, Oberkochen, Germany) equipped with a 55 Axiocam 503 colour camera). The glass chamber was connected to an automated gas delivery system assembled in-house that alternately pushed the VOC sample or clean air (or nitrogen with 50% relative humidity) through the chamber in a cyclic way. A cycle consists of an exposure stage (VOC is pushed into the chamber) and a recovery stage (clean air or humid nitrogen are pushed into the chamber to remove the VOC). The alterations in the LC order within the droplets were visible in real-time and video-recorded with a 1936 × 1460 resolution using the microscope camera. For the VOC classification task, exposures to samples of 11 VOCs (acetone, acetonitrile, chloroform, dichloromethane, diethyl ether, ethanol, diethyl acetate, heptane, hexane, methanol, toluene) were recorded. To generate the VOC samples, the gas delivery system pushed air through the headspace of a 30 mL vial containing 15 mL of pure solvent at 37 °C (saturated atmosphere conditions). The concentration of VOC in the sample was between 12% and 15% (*v/v*), estimated as explained in a previous publication [20]. Each gel was subjected to 5 consecutive exposure/recovery cycles with one VOC. There were 11 gels and, in total, 55 cycles were performed. The videos were recorded in a single region of the gel and had 32 frames of coloured images (RGB). For the VOC concentration prediction task, we used acetone as model VOC. Acetone can interact with the different components of the gel and promote rich optical textures patterns. A gas delivery system was assembled to generate concentrations of acetone between 0.9% and 4.0% (*v/v*). Acetone vapours were generated by bubbling nitrogen through pure acetone. To obtain the different concentrations, two mass flow controller devices (MFC) were used to mix humid nitrogen and acetone vapours in different proportions (Figure 2). Nitrogen was pumped by the dilution MFC and bubbled through water to generate 50% relative humidity in the stream. Acetone vapours were pumped by the carrier MFC. By keeping the dilution MFC flow rate constant (5 L/min) and varying the carrier MFC flow rate (0.2, 0.28, 0.36, 0.44, 0.52, 0.6, 0.68, 0.76, 0.84, 0.92 and 1 L/min), increasing concentrations of acetone vapours in the humid nitrogen stream were obtained (estimated 0.9%,1.3%,1.6%,2.0%,2.3%,2.6%,2.9%,3.2%,3.4%,3.7% and 4.0% (*v/v*) [25]) and sent to the sensor chamber (see Figure 2). Two independent gels were exposed to a sequence of exposure/recovery cycles with the 11 increasing concentrations of acetone. Videos were recorded in three distinct regions of each gel and had 78 frames of coloured images (RGB). Thus, 6 cycles were recorded for each concentration. Overall, 66 cycles were performed.

### 2.3. Video Analysis Strategy

**Figure 3:**
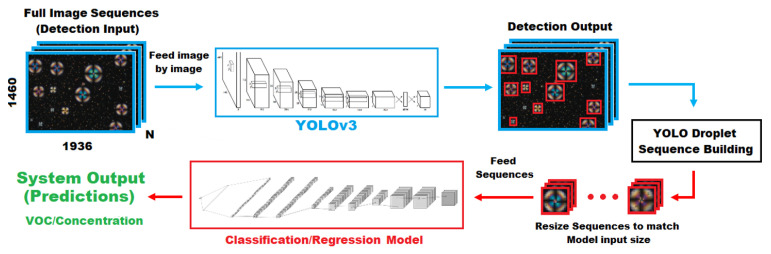
Full architecture of video analysis system. YOLOV3 is responsible for detecting the droplets. These droplets are assembled into sequences and resized prior to being fed to the classification or regression model.

A large number of droplets can be observed in our videos (Figure 1b). These droplets and their evolution through time (Figure 1c) are the subjects of our analysis. Therefore, we start by detecting all the droplets in each video and by creating full cycle sequences for individual droplets. Since droplets have varying diameters, it is then necessary to resize the droplets image sequences to a common diameter. After that we extract features from the video sequences with different 2D and 3D CNN approaches. Finally, a classification or regression task is performed. Figure 3 presents a scheme of the overall system architecture. The following sections detail each of the steps.

#### 2.3.1. Droplet Detection

For droplet detection, we used YOLO-v3 [26], a state of the art deep learning architecture designed for fast object detection and classification, which divides images into regions and then predicts bounding boxes and class probabilities for each region. After non-maximal suppression, YOLO will provide an output with all the detected bounding boxes and respective classes. In this work there is only one class: the droplet. The YOLO network produces bounding boxes around droplets on single images and not on 3D images/image sequences. Therefore, we obtained droplet bounding boxes for every frame and then assembled them into sequences according to the following criteria. For pairs of bounding boxes in the same frame, the intersection over union (IoU), computed as the area of intersection divided by the area of overlap was computed. IoU scores over 0.3 were considered to be detecting the same droplet (it may happen despite non maximum suppression) and only the one with greater area is kept. Bounding boxes from the first frame are set as reference. Then, every bounding box in the next frames that achieves an IoU score above 0.5 with one of the reference ones is associated with it, meaning that it is very likely to be circumscribing the same droplet on a different frame. After this association, sequences that contain very few bounding boxes, less than 25% of the sequence total number of frames, were also discarded. Finally, the mean bounding box coordinates in the entire sequence are computed to obtain the sequence width and height.

#### 2.3.2. Feature Extraction

For feature extraction we use CNNs [27], which are the most widely used deep learning models for image and video applications. Two different CNN architectures were tested for feature extraction. One is a full 3D CNN, where the third dimension is time, and the other one combines a 2D CNN applied to every image in the sequence with a Long Short Term Memory (LSTM) [28] which is a type of recurrent neural network that extracts time domain information.

**Figure 4:**
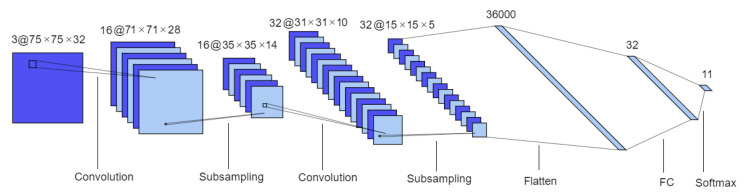
3D CNN architecture. Format: number of channels or filters @ image dimension. This model is composed of two sets of convolutional and subsampling layers followed by two fully connected layers.

The 3D CNN is inspired in the Lenet-5 model [29]. It is also composed by two sets of convolutional and MaxPooling layers followed by two fully connected layers. However some changes were introduced, namely ReLU activation functions were used instead of tanh, and a batch normalization layer was introduced between each convolutional layer and its activation function, as well as a dropout layer immediately after. Additionally, Lenet-5 inputs and convolutional filters (kernels) are 2D whereas in this network they are 3D. This architecture is represented in Figure 4.

**Figure 5:**
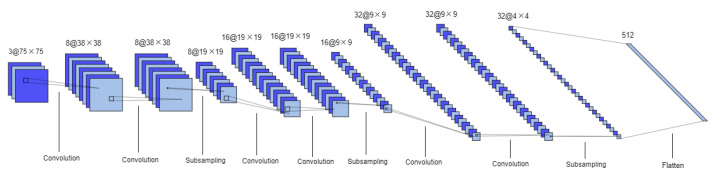
2D CNN architecture. This CNN is composed of three convolutional blocks, each with two convolutional layers and a MaxPooling layer, followed by a flattening layer.

The 2D CNN is a deeper model composed by three sets of two convolutional layers (with ReLU activation) and a MaxPooling layer, totaling 9 layers. Padding is applied in the convolutional layers and there is a dropout layer before the final classification or regression layer (output layers are described in the next section). This architecture is illustrated in Figure 5.

The 2D CNN is applied to every frame in the image sequence (the same CNN), extracting features that are then fed to an LSTM which will learn time features for each sequence. This LSTM is of the type “Many to One” since its input is a sequence of features and the output is a single value, obtained through the returned values of the last cell, which will subsequently be used to predict a VOC class or concentration. The amount of cells that compose the LSTM is the same as the sequence length.

**Figure 6:**
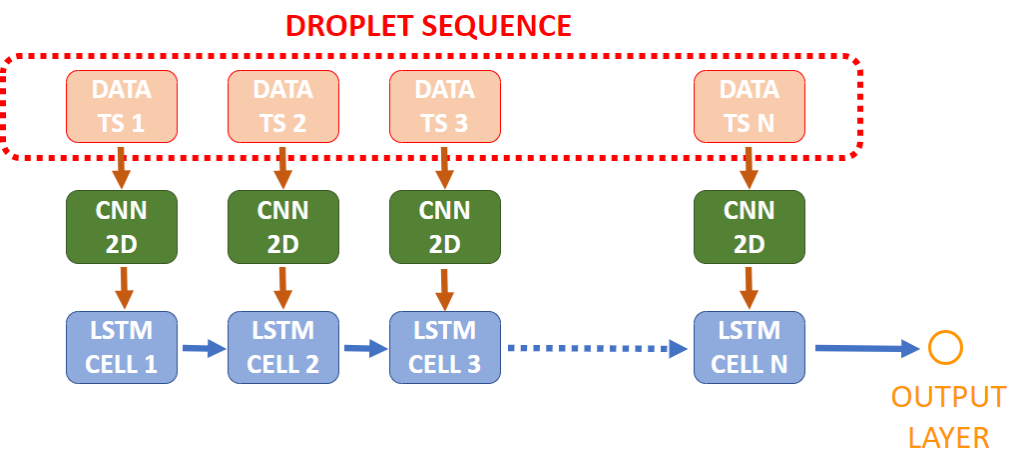
LSTM + CNN2D architecture scheme. The CNN represented by the green boxes is the same applied to every frame in the sequence. TS denotes time step. Each time step corresponds to a different frame in the sequence.

Figure 6 presents a scheme on how LSTM and 2D CNN models are combined.

#### 2.3.3. Classification and Regression

Depending on which task the models were applied to, the last layer in the neural network was either a 11 unit softmax activation function (for 11 VOC Classification) or a linear unit, which is a single neuron without any activation function (for concentration regression).

**Figure 7:**
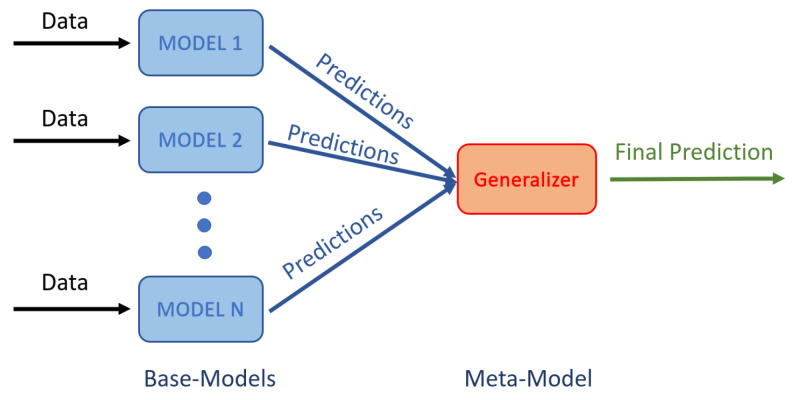
Architecture of the stacking ensemble: Several base classification models are applied to the same data and the meta model learns how to best combine the predictions from the different base models.

Additionally, a stacking ensemble [30] was used to combine the predictions obtained from a number of base models from each architecture. Each base model was of the same type (CNN3D or CNN2D+LSTM) but trained using a different combination of parameters such as the number of filters, filter sizes, amount of dropout and number of units in the fully connected layers. The meta model aggregates the base models outputs to produce the final prediction, as illustrated in Figure 7. The Gaussian Naive Bayes classifier was used as meta-model.

#### 2.3.4. Performance Evaluation

CNN models for VOC classification are evaluated using accuracy and F1-score, for each VOC and globally using macro F1-score. Accuracy is the fraction of the total samples that were correctly classified which is computed as:

(1) $$\mathrm{Accuracy}=\frac{\mathrm{TP}+\mathrm{TN}}{\mathrm{TP}+\mathrm{FP}+\mathrm{TN}+\mathrm{FN}}$$

For each VOC, we consider TP (true positives) as the number of correctly classified samples from that VOC and TN (true negatives) as the number of correctly classified samples from all the other VOCs. Similarly, FP (false positives) is the number of incorrect classifications of a given VOC and FN (false negatives) corresponds to the combined number of missed detections from all the other VOCs.

The per VOC F1-score is the harmonic mean between its Precision and Recall, computed as:

(2) $$F1=2\times \frac{\mathrm{Precision}\times \mathrm{Recall}}{\mathrm{Precision}+\mathrm{Recall}}$$

where Precision is the proportion of correctly classified samples of a given VOC out of all samples classified as that VOC, computed as:

(3) $$\mathrm{Precision}=\frac{\mathrm{TP}}{\mathrm{TP}+\mathrm{FP}}$$

and Recall is the proportion of correctly classified samples of a VOC out of all the samples from that particular VOC, computed as:

(4) $$\mathrm{Recall}=\frac{\mathrm{TP}}{\mathrm{TP}+\mathrm{FN}}$$

The macro F1 is the average (unweighted) of the F1 score obtained for each VOC.

CNN models designed to estimate concentration are evaluated using mean absolute error (MAE), specifically:

(5) $$\mathrm{MAE}=\frac{1}{n}\sum_{k=1}^{n}\left|e_{k}\right|$$

where $e_{k}$ represents the difference between true concentration and predicted one for droplet *k*, and *n* is the total number of droplets.

YOLO models for droplet detection are evaluated using average precision (AP) which is the most commonly used metric to evaluate the performance of object detection algorithms. The implementation provided in [26] for mAP calculation was used. In this case, mAP and AP are the same since there is only one type of object to detect.

## 3. Results

This section presents the results obtained in both tasks, VOC recognition and acetone concentration prediction, as well as an analysis on the impact of droplet diameter on these results.

For the VOC recognition task, the 3 first exposure cycles from each VOC were selected for the training set, the fourth cycle for the validation set and the last one for the test set. Every cycle is kept in the form of an image sequence formed by 32 RGB frames with 1936 × 1460 pixels each.

For the acetone concentration task, four of the exposure cycles were selected for the training set, one for the validation set and remaining one for the test set. In this case, every exposure video is composed by 78 RGB frames with 1936 × 1460 pixels.

In both tasks, the training set is used to train the neural networks, the validation set is used to select the best hyper-parameters and avoid overfitting by means of early stopping, and the test set is used only to evaluate performance.

### 3.1. Droplet Detection

The YOLO network used to perform the droplet detection was an implementation made available on GitHub (https://github.com/wizyoung/YOLOv3_TensorFlow, accessed on 5 January 2021).

Ground truth annotations were manually created for droplets bigger than 12 μm × 12 μm. With the set of parameters in Table 1, and after the model loss stopped decreasing on the validation set, training was stopped.

**Figure 8:**
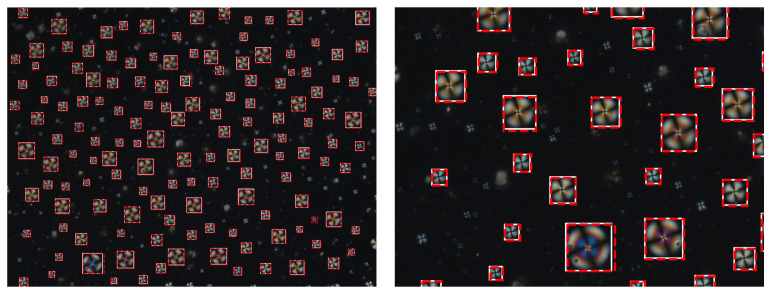
Droplet Bounding Boxes. Ground truth bounding boxes are shown in white whereas YOLO detection are shown in red. (**Left**) whole Frame; (**Right**) zoomed in section.

On the VOC recognition test set YOLO was able to achieve an AP of 0.94. Figure 8 illustrates the detection outputs. This figure shows an image from the test set, ‘unseen’ by the model, where the YOLO bounding boxes are represented in red and the ground truth bounding boxes are shown in white.

On the acetone concentrations test set, YOLO achieved an AP of 0.87. After the YOLO frame by frame detections were obtained, they were assembled into sequences resulting in the following number of sequences in each set (Table 2).

Since CNN models have fixed input sizes and the droplets have a wide range of diameters, all images in each sequence were resized to a common size of 75 × 75 pixels before being used in the subsequent tasks.

### 3.2. VOC Recognition

For classification, 3 dimensional CNNs (CNN3D) and LSTMs with 2 dimensional CNNs (CNN2D+LSTM) working as feature extractors were used and a softmax was used as output layer. The parameters of these networks are presented in Table 3. To select the hyper-parameters a grid search is performed and the set that achieves best overall performance in the validation set is selected. The ensembles of CNN-3D used 9 base models and the ensembles of CNN2D+LSTM used 12 base models, corresponding to the total number of combinations of different hyper-parameter values. Every model was trained with the ‘Adam’ optimizer and using Categorical Crossentropy as the loss function.

The results obtained with single and ensemble models are presented in Table 4.

**Figure 9:**
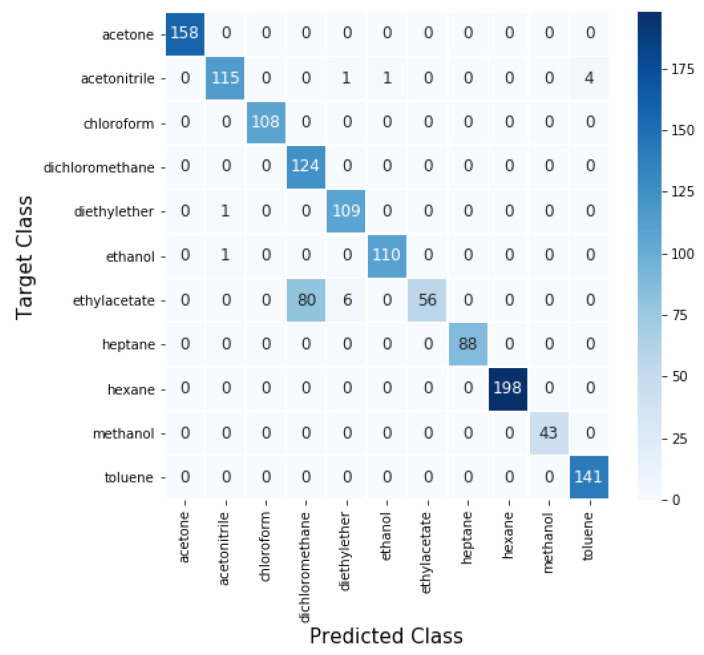
Confusion matrix for CNN2D + LSTM ensemble. Values in the diagonal represent correct predictions and off diagonal values represent incorrect predictions.

For the best model, corresponding to the CNN2D+LSTM ensemble, the confusion matrix with the prediction results obtained in the identification of the 11 VOC, is represented in Figure 9.

**Figure 10:**
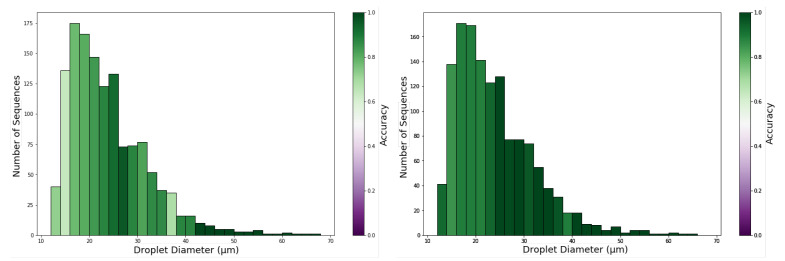
Test set accuracy by droplet diameter for ensemble models. (**Left**) CNN3D ensemble; (**Right**) CNN2D+LSTM ensemble.

To evaluate the impact of droplet diameter on the performance of VOC detection, we plot in Figure 10 an histogram of droplet diameters where the bin colour represents the accuracy in predictions of the droplets in that diameter range.

### 3.3. Acetone Concentration

For this task, the same CNN architectures were used for feature detection but a linear unit was used in the output layer. Instead of the Categorical Crossentropy, the Mean Squared Error loss function was used since this is now a regression problem. The optimizer was again ‘Adam’.

**Figure 11:**
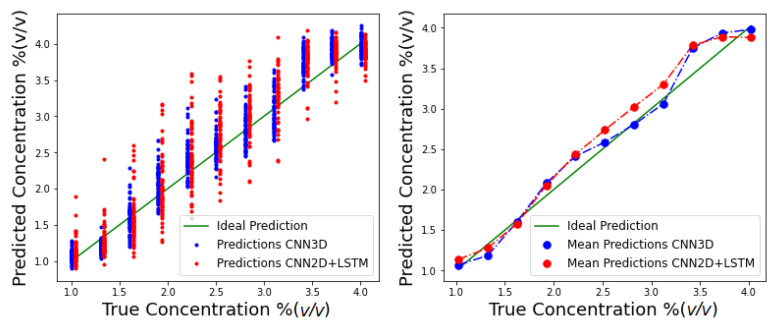
CNN3D and CNN2D+LSTM test set concentration predictions. (**Left**) individual droplet predictions; (**Right**) average predictions.

The predictions of individual YOLO droplet sequences in the test set are plotted in Figure 11, along with average predictions.

From these predictions the following mean absolute errors were obtained (Table 5).

**Figure 12:**
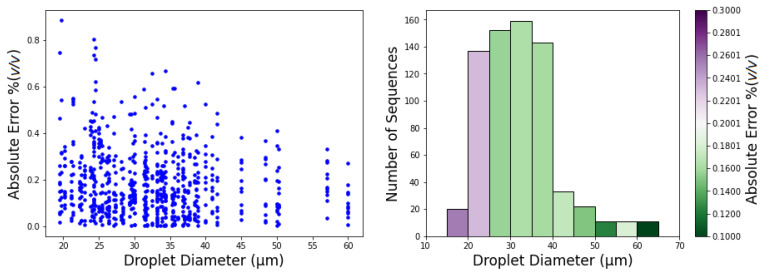
CNN3D test set MAE as a function of droplets diameters. (**Left**) individual droplet prediction error; (**Right**) MAE by droplet diameter.

Regarding the influence in performance of the droplet diameter, Figure 12 presents the results that were obtained for MAE as a function of droplet diameter.

## 4. Discussion

### 4.1. YOLO Detection

The YOLO network was able to detect droplets in individual frames with high average precision. It obtained an AP of 0.97 for the VOC classification test set and 0.87 for the concentrations test set. The bounding boxes that were obtained are very close to the ground truth ones, perfectly encapsulating the droplet, as illustrated in Figure 8. The majority of false detections occurred for very small droplets that were not included in the manually annotated ground truth.

### 4.2. VOC Recognition

F1-scores obtained for the classification of 11 VOC are shown in Table 4, for both single models and corresponding ensembles. The best macro F1-score 0.932 was obtained for the CNN2D+LSTM ensemble. For single models, CNN2D+LSTM outperformed CNN3D, with macro F1-scores of 0.901 and 0.748, respectively. Both ensembles performed better than corresponding single models, with improvements for almost every VOC. The average improvements were nearly 10% in the case of CNN3D and around 3% for the CNN2D+LSTM. This suggests that the best hyper-parameters that were chosen for the single models are not the most suitable for every VOC or every droplet diameter. The selected 11 VOCs present small structural and functional differences, including alkanes, ketones, alcohols, aromatics and chlorinated VOCs. Due to their distinct chemical properties, the selected VOCs interact preferentially with distinct components of the gel. For example, hydrophobic compounds (such as heptane, hexane, toluene) are likely to interact with the LC inside the droplets, while those that can form hydrogen bonds (such as ethanol, methanol, acetone) preferentially interact with the biopolymer matrix, but also with the ionic liquid and liquid crystal. As a result of the distinct interactions, LC orientational changes happen in different timings and create distinct dynamic optical texture variations (see Supplementary Videos S1 and S2), as previously described in the team’s past works [19,20]. Our results show that the pattern of those variations represents VOC fingerprints since the implemented models achieved excellent VOC classification performances.

To analyze the impact of droplet diameter on the quality of predictions, an histogram of droplet diameters for the test set was plotted in Figure 10 where the bin colour represents the accuracy obtained in the classification of the droplets in that diameter range. This figure shows that the number of droplets decreases with increasing diameter, but that with an increase in diameter, there is also an increase in accuracy. While sequences of smaller droplets (<20 μm) only achieve accuracies below 70%, with the increase in diameter, the accuracy increases near to 100%. Taking in consideration the number of sequences available for each droplet diameter range, the results indicate VOC recognition task is optimised by droplets with diameter larger than 24 μm and using the CNN2D+LSTM architecture (Figure 10). This might be associated with richer colour palettes and response dynamics from larger droplets compared to smaller ones. Furthermore, 24 μm is close to the 30 μm thickness of the gel. Droplets with diameter smaller than 24 μm might be embedded deeper in the gel matrix than the larger ones and as such, the number of molecules of VOC that reaches the LC may be limited and insufficient to promote LC orientational transition patterns sufficiently distinct between different VOCs.

Regarding individual VOC recognition, several architectures perfectly classified some of the VOCs, like acetone, hexane and methanol and heptane is even perfectly classified by all the models. Diethyl acetate and dichlorometane are the hardest VOCs to classifiy across architectures, in fact for the best model (CNN2D+LSTM ensemble) the worst results are registered with diethyl acetate which achieves an F1-score of only 0.594, followed by dichlorometane with an F1-score of 0.763. This is related with the diameter of droplets exposed to diethyl acetate which, in the majority of cases, is under 24 μm. Since smaller droplets are harder to classify this affects the performance obtained for this VOC. It also affects the performance of dichlorometane because most of the miss-classified diethyl acetate sequences were wrongly classified as dichlorometane, as shown in the confusion matrix from Figure 9.

### 4.3. Acetone Concentration

Predicted concentration values obtained by CNN3D and CNN2D+LSTM on the test set are presented in Figure 11. It is possible to observe that despite a large variance, estimates from both models are mostly centered around the true value and consequently the mean concentrations are close to the real ones for both architectures, particularly for lower values of the flow. The best MAE (0.2425% (*v/v*)) was obtained by the CNN3D but CNN2D+LSTM was close with a MAE of 0.2669% (*v/v*) (Table 5). Acetone molecules can interact with all the hybrid gel components, for example by hydrogen bonds with the gelatin matrix and ionic liquid, and by dipole-dipole interactions with the liquid crystal molecules. Thus, when the gel is exposed to increasing concentrations of incoming acetone molecules, the number of interactions with the gel components increases and ultimately promotes a gradual increase in the number of LC molecules that undergo orientational transitions. As a result, there is a relation between the optical texture variation patterns and the acetone concentration, that is captured by the implemented regression model.

Regarding the impact of droplet diameter on these results, the histogram of droplet diameters shown in Figure 12 reveals that the large majority of droplets has diameter between 20 μm and 40 μm. From the plot of droplets error and from the bin colors in the histogram, which represent the MAE, we observe a tendency of bigger droplets to obtain better (lower MAE) concentration estimates. The exception is in the penultimate bin (<60 μm) where the error is higher, a variation which is interpreted as due to the small samples size.

## 5. Conclusions

In this work we explored a VOC pattern recognition approach which is fully automated and takes advantage of the full extent of information carried by LC optical textures, namely morphological and colour changes along time. In our approach, CNN were used to analyse the optical textures dynamics in LC upon exposure to a set of 11 distinct VOCs containing compounds with very small structural differences but from distinct chemical groups (acetone, acetonitrile, chloroform, dichloromethane, diethyl ether, ethanol, diethyl acetate, heptane, hexane, methanol and toluene). From the video analysis of individual LC droplets, the proposed system was able to accurately classify the 11 VOCs (F1-score higher than 93%). In a separate task, the system was used to predict the concentration of acetone vapours exposed to the gel and achieved mean absolute errors under 0.25% (*v/v*). These results confirm the rich information content carried by optical textures of LC droplets in hybrid gels, and show that video analysis is an alternative to 1D optical signal analysis for VOC sensing. Individual droplets respond fast (in less than 20 s) and are sufficient for VOC discrimination and quantification. Furthermore, the hybrid gel production method is simple and scalable [19] and so are the proposed CNN models. Therefore, the system has potential to be implemented in the form of a miniaturised gas delivery system and optical detection device based, for example, on the currently available advanced smartphone cameras. Such systems contribute to the development of small, fast and sustainable gas sensing devices.

## Figures and Tables

**Table 1:** YOLO parameters during training.

Parameters	Value
Batch Size	5
Image Resize	416 × 416
Keep Aspect Ratio	True
Batch Norm Decay	0.99
L2 Weight Decay	5 × 10${}^{-3}$
Optimizer	Momentum
Learning Rates	1 × 10${}^{-3}$, 3 × 10${}^{-4}$, 1 × 10${}^{-5}$
Fine Tuning	Whole Model
NMS Threshold	0.5
mAP Threshold	0.5

**Table 2:** Number of droplet sequences in each data set.

Task	Train	Validation	Test
VOC recognition	4295	1368	1345
Acetone concentration	4513	920	699

**Table 3:** CNN Parameters. Hyper-parameters are shown in bold.

Parameters	CNN3D	CNN2D + LSTM
Number of Filters	16, 32	8, 16, 32
Filter Dimensions	**(** 10×10×10 **), (** 10×15×15 **), (** 10×20×20 **)**	**(** 3×3 **), (** 5×5 **), (** 7×7 **)**
Stride	1	1
Max-Pool window	2×2×2	2×2
Max-Pool stride	2	2
Dropout Rate	**0.2, 0.35, 0.5**	0.5
Units FCL 1	32	**16, 32**
Units LSTM	-	**16, 32**
Units Softmax	11	11

**Table 4:** VOC recognition F1-score on test set. The best macro F1-score is shown in bold.

	Single Models		Ensemble Models	
VOC	CNN3D	NN2D+LSTM	CNN3D	CNN2D+LSTM
Acetone	1	0.981	1	1
Acetonitrile	0.569	0.887	0.732	0.962
Chloroform	0.807	0.977	1	1
Dichloromethane	0.167	0.834	0.467	0.763
Diethyl ether	0.876	0.803	1	0.96
Ethanol	0.965	0.978	0.972	0.991
Diethyl acetate	0.465	0.608	0.191	0.594
Heptane	1	1	1	1
Hexane	0.978	0.990	1	1
Methanol	0.951	0.989	1	1
Toluene	0.451	0.940	0.889	0.982
**Macro**	0.748	0.908	0.841	**0.932**

**Table 5:** Test set MAE for the two CNN architectures used to predict acetone concentration.

Model	MAE% (*v/v*)
CNN3D	0.2425
CNN2D+LSTM	0.2669

## Data Availability

Data and code used in this work are available on Github: https://github.com/canion8/optical_Gas_Sensing_with_CNN (accessed on 18 April 2021).

## Supplementary Materials

The following are available online at https://www.mdpi.com/article/10.3390/s21082854/s1, Video S1: Optical textures variation in a hybrid film during five exposure/recovery cycles with acetone, Video S2: Optical textures variation of a single acetone droplet.

## Abbreviations

The following abbreviations are used in this manuscript:

AP	Average precision
CNN	Convolutional neural network
DMMP	Dimethyl-methylphosphonate
FN	False negatives
FP	False positives
IOU	Intersection over union
LC	Liquid Crystal
LSTM	Long short time memory
MAE	Mean Absolute Error
mAP	Mean average precision
MFC	Mass flow controller
NMS	Non maximum suppression
POM	Polarised optical microscope
ReLU	Rectified linear unit
RGB	Red Green Blue
SLPM	Standard litre per minute
SVM	Support vector machine
VOC	Volatile organic compound
TN	True negatives
TP	True positives
TS	Time step
YOLO	You only look once
